# Surgical Anatomy

**Published:** 1850-10

**Authors:** 


### BIBLIOGRAPHICAL NOTICES.

---

*Surgical Anatomy.* By JOSEPH MACLISE, Surgeon. *With colored plates.* Part III. To be completed in four parts. Philadelphia: Lea & Blanchard: 1850.

The 1st and 2d parts of this work have already gained for it a high reputation as a treatise upon Surgical Anatomy: it is, therefore, unnecessary to give any detailed account of the contents of this part, in order that we may commend it to our readers. Every one who is conversant with the medical literature of our country, is conscious that there is an imperative demand for works of this class, and will congratulate the American public upon the appearance of Mr. Maclise's labors in their present form and dress.

The importance of this kind of knowledge, the difficulties under which it is usually obtained, and the facility with which it is forgotten, afford cogent reasons for pressing the usefulness and value of this work upon every student of medicine.

Although we cannot admit for one moment that there is any authority in Anatomy equal to the dead subject and the scalpel, and that for these there can never be a substitute, nevertheless, since so few appeal to this source of knowledge, and since (of these few) some approach it with preconceived notions from books; we cannot but rejoice that there are some sources of authority that are in accordance with nature and the truth. If a large number *will* teach Anatomy from books, and a still larger number learn it from the same source, we rejoice to know that Maclise's Surgical Anatomy will inculcate more truth and fewer errors than most of its predecessors.

This 3d part contains sixteen plates relative to Inguinal and Femoral Hernia, with instructive commentaries upon each. We hail those upon Inguinal Hernia with most pleasure, because we conceive that greater error exists among teachers and students upon this form of hernia than upon any other. Every one, whether an Anatomist or Surgeon, must recollect the time and labor expended upon this subject in the lecture-room, and the apparent paradoxes and contradictions with which it was surrounded. The very fact, that the subject of Hernia is a "pons asinorum" to students, is an evidence that there is an obscurity connected with its demonstration. The parts concerned are not so numerous or minute as those of the eye or brain, and yet these latter are learned with comparatively little patience and trouble. Though the points to be studied in hernia are few in number, and can be seen without microscopes, the student generally leaves the lecture-room with a degree of perplexity which cannot in justice be attributed to the subject, and which is only removed by his own personal dissections.

This obscurity arises from the application of a defective nomenclature, a kind of adherence to the description given by some great authority, and in making dissections to *suit a description.*

Anatomical nomenclature probably never can be altered: we can never expect a perfect uniformity of terms, nor is it necessary that there should be, any more than that we should all speak the same language. But, it is extremely important that students should be taught something more than mere names, that they should not be confused with long Latin synonymes, before receiving clear ideas of facts: they should learn that terms are but conventional and representative, whether invented by Camper, Scarpa, or Sir Astley Cooper. These are the views and feelings of Mr. Maclise when he speaks of the difficulty and perplexity with which this impenetrable fog of surgical nomenclature besets the progress of the beginner.

We have no hesitation in saying that no one is able to make a proper demonstration of the anatomy of hernia, unless he can make an intelligent beginner clearly understand it without the use of technical terms.

How often has an hour been wasted in endeavoring to prove that Sir Astley Cooper called this or that the Cribriform Fascia; as if the great idea to be taught was, that the dissection presented to their view was in accordance with that of Sir Astley; as if the truth was not the same, no matter upon what plan the dissection is made, provided it is not forced by knife-handles or pointers.

How unfortunate would it be for a student if this fond adherence to terms should affect the prescribed text-books of any medical school; one with an air of confidence and certainty will say, this is the "cribriform fascia," another with a tenacity equal to the other's boldness, "that is the cribriform fascia; Sir Astley told me so."

"Strange there should such a difference be
Twixt tweedle dum and tweedle dee."

A sentiment which seems to have actuated Mr. Maclise in his work is thus expressed by him : " But when we view nature as she is, and not fashioned by the scalpel, we never fail to find an easy explanation of her form." This follows a description of the inferior edges of the internal oblique and transversalis muscles. Our readers may recall, among the dim memories of inguinal hernia, something, called the crural arch, and, perhaps, they may recollect seeing the same pictured forth with beautiful regularity, in some drawing or painting of the subject, about the lower part of the belly.

Now, Mr. Maclise says nothing about the crural arch in this connexion, and hence, therefore, some may not consider the work as orthodox ; especially as they *know* that Colles, or Scarpa, or Cloquet, or Lawrence, says there is a crural arch. This reference to authority and not to the dead subject, at once settles the matter in their minds. But let the reader peruse the following passage, and judge for himself.

" The arched inferior border of the transverse muscle, F, Plate 30, expresses by its abrupt termination that some part is wanting to it ; and this appearance, together with the fact that the fibres of this part of the muscle blend with those of the internal oblique and cremaster, and cannot be separated except by severing the connexion, at once, suggests the idea that the cremaster is a derivation from both of these muscles.

" Assuming this to be the case, therefore, it follows that when the dissector removes the cremaster from the space L *h*, he himself causes this vacancy in the muscular parietes of the groin to occur, and at the same time gives unnatural definition to the lower border of the transverse and oblique muscles. In a dissection so conducted, the cord is made to assume the variable positions which anatomists report it to have in respect to the neighboring muscles. But when we view nature as she is, and not as fashioned by the scalpel, we never fail to find an easy explanation of her form.

" In the fœtus, prior to the descent of the testicle, the cremaster muscle does not exist. (Cloquet. op. cit.) From this we infer, that those parts of the muscles, EF,* Plate 30, which at a subsequent period are converted into a cremaster, entirely occupy the space L *h*. In the adult body, where one of the testicles has been arrested in the inguinal canal, the muscles E F, do not present a defined arched margin, above the vacant space L *h* but are continued (as in the fœtus) as low down as the external abdominal ring. In the adult, where the testicle has descended to the scrotum, the cremaster exists, and is serially continous with the muscles E F, covering the space L *h* ; the meaning of which is, that the cremasteric parts of the

* Internal, oblique and transversalis.

muscles E F, cover this space. The name cremaster therefore must not cancel the fact that the fibres so named are parts of the muscles E F. Again, in the female devoid of a cremaster, the muscles E F present of their full quantities, having sustained no diminution of their bulk by the formation of a cremaster. But when an external inguinal hernia occurs in the female body, the bowel, during its descent, carries before it a cremasteric covering at the expense of the muscles E F, just in the same way as the testicle does in the fœtus. (Cloquet.)

"From the above-mentioned facts, viewed comparatively, it seems that the following inferences may be legitimately drawn :—1st, that the space L *h* does not naturally exist devoid of a muscular covering; for, in fact, the cremaster overlies this situation ; 2d, that the name cremaster is one given to the lower fibres of the internal oblique and transverse muscles which cover this space ; and 3d, that to separate the cremasteric elongation of these muscles, and then describe them as presenting a defined arched margin, an inch or two above Poupart's ligament, is an act as arbitrary on the part of the dissector as if he were to subdivide these muscles still more, and, while regarding the subdivisions as different structures, to give them names of different signification. When once we consent to regard the cremaster as constituted of the fibres originally proper to the muscles, E F, we then are led to the discovery of the true relations of the cord in respect to these muscles."

There is another point upon which we consider Mr Maclise to have added much that is valuable to teachers and students, and this is expressed in plates 31 and 33, exhibiting the fascia transversalis as far as it relates to the anatomy of parts concerned in hernia, a point concerning which there is such a diversity of expression.

This diversity of opinion arises, as we have said already, from a want of reference to the dead subject, and a dependence upon authority for truths which we should acquire or make our own by study. Let the reader refer for instance to the plate of Sir A. Cooper exhibiting his fascia transversalis, and that which is called the internal abdominal ring, and he will there see represented a dissection which is well calculated to give a most erroneous idea, and which is a most fruitful source of error on this subject. There is pictured an oval hole in the fascia which was made by the dissector, and which is understood by the ignorant to be so in nature, which Sir A. C. never meant to teach. In this point and in many others we consider Mr. Maclise's drawing far superior to any others, for the simple reason that it represents the truth.

The " internal spermatic fascia," (Cooper) and "infundibuliform fascia," (Cloquet) is shown to be continuous with, but a prolongation of, the fascia transversalis. This is not only true in nature where no hernia exists, but also where inguinal hernia does occur, unless the case is an exception, and the hernia is the result of force and the tissue actually ruptured. If the work of Mr. Maclise be read and studied by those who cannot or will not read nature, they cannot fail to get rid of the erroneous impressions which result from early education, and learn that the internal abdominal ring is not a hole or fenestrum.

The plates illustrating the descent of the testicle are equally well calculated to teach the truth as it is in nature, and the laws by which she is governed. They show how the testicle descends, pushing before it, and enveloping itself with, every lamina which constitutes the parietes of the abdomen; the modifications which these laminæ undergo in becoming the investments of the cord and testicle; and that in every oblique inguinal hernia, the bowel pursues the same course.

The work is equally satisfactory with reference to Femoral Hernia. The plate exhibiting the crural ring and the description, the descent of the bowel and the manner in which it receives its coverings, are shown in a clear and instructive manner; and we have no doubt that many will in their hearts confess that this work of Mr. Maclise has taught them what they never knew before concerning the anatomy of hernia.

How simple does the subject of hernia become when treated of by a philosophical anatomist, who is not only familiar with facts and details, but with the causes which produced them! How indelible is the truth acquired by reason compared with that acquired by memory. When in the first place we are taught the true anatomy of the parietes of the abdomen in general, and then the descent of the testicle, and the particular relation of these parietes to the cord and testicle, and that when the bowel descends, it follows the course of the cord (in oblique inguinal hernia,) and that whatever is true of the cord and its coverings, is true of the protruded bowel,—we then distinctly see the reason of things, and the connection of one fact with another, that *rupture is not a rupture;* that this term is calculated to give rise to the idea of holes with defined edges; whereas, there is no such thing in nature, not even at the external abdominal ring; and whenever such an opening is presented to us, we recognize it at once as the result of an injury, or as a false dissection.

Such works as this are calculated to bring about the correct teaching of a *Philosophical Anatomy.*

We extend to this number, therefore, as we have to those which preceded it, a hearty welcome, and commend it to our readers as a faithful exponent of the science of which it professes to treat.

Nothing will be believed now, but that which one can see for himself; and fortunately the laborers in this field are sufficiently numerous to present the science of anatomy in a new light, with no claims but those based simply on facts and their philosophy.

